# The complete mitochondrial genome of *Apis nuluensis* Tingek, an Asian honey bee (Insecta: Hymenoptera: Apidae)

**DOI:** 10.1080/23802359.2017.1365655

**Published:** 2017-08-17

**Authors:** Amin Eimanifar, Rebecca T. Kimball, Edward L. Braun, Stefan Fuchs, Bernd Grünewald, James D. Ellis

**Affiliations:** a Entomology and Nematology Department, Honey Bee Research and Extension Laboratory, University of Florida, Gainesville, FL, USA;; b Department of Biology, University of Florida, Gainesville, FL, USA;; c Institut für Bienenkunde, Polytechnische Gesellschaft, Goethe-Universität Frankfurt am Main, FB Biowissenschaften, Oberursel, Germany

**Keywords:** Cavity nesting honey bee, mitogenome, next generation sequencing, *Apis nuluensis*, Southeast Asia

## Abstract

The complete mitochondrial genome of *Apis nuluensis* Tingek was sequenced. The mitochondrial genome was 15,843 bp in length, with 37 classical eukaryotic mitochondrial genes and an A + T-rich region. Gene directions and arrangements were similar to those of other *Apis* mitogenomes. Most genes initiate with ATT (though ATG and ATC also were used) and all genes terminated with TAA. Nine genes were encoded on the light strand while four were encoded on the heavy strand. All 22 tRNA genes have a typical cloverleaf structure. The most likely phylogenetic tree showed *A. nuluensis* clustering with *A. cerana*. The complete mitogenome of *A. nuluensis* completes the sequencing of all mitogenomes of the currently accepted species of *Apis*.

*Apis nuluensis* Tingek is honey bee species endemic to the montane areas of the Mt. Kinabalu regions in northern Borneo (Tanaka et al. 2001). *Apis nuluensis* is a cavity-nesting honey bee that is morphologically and behaviourally distinct from other sympatric species, *A. cerana* Fabricius, *A. koschevnikovi* Enderlein, and *Apis nigrocincta* Smith (Tingek et al. 1996). An adult honey bee worker of *A. nuluensis* was obtained from the Ruttner Bee Collection at the Bee Research Institute in Oberursel, Germany (Voucher no. 1929, Malaysia, Borneo Sabah, 5°58N, 116°20E) and its mitogenome was reported (GenBank Accession No. MF565375). The individual was identified using discriminant analysis for multivariate morphological allocation to species by Institute staff. Extraction of total genomic DNA and quantifications were performed as described in Eimanifar et al. (2016). A genomic library was constructed from the genomic DNA using a Kapa Hyper Prep Kit (Kapa Biosystems, Woburn, MA) with a paired-end read (2 × 150) followed by next generation sequencing technology on the Illumina Hi-Seq 3000/4000 (San Diego, CA). The mitochondrial genome assembling and annotation were performed as described in Eimanifar et al. (2017). The assembled mitogenome was aligned with those of other *Apis* spp. using Mesquite v 3.10 (Maddison and Maddison 2016) and manually adjusted. Based on examination of the sequences, putative pseudogenes were present for some regions of the mitochondrion.

The complete sequence of *A. nuluensis* was 15,843 bp in length and consisted of 13 protein-coding genes, 22 transfer RNA (tRNA) genes, two ribosomal RNA (rRNA) genes, and one putative control region (CR). The overall base composition of the *A. nuluensis* mitogenome was A (42.0%), T (41.8%), C (9.9%), and G (6.2%), respectively. The gene content, structure, and arrangement of the *A. nuluensis* mitogenome were similar to those observed in other *Apis* spp. mitogenomes (Eimanifar et al. 2016, 2017). Four mitochondrial genes were encoded on the H-strand and nine on the L-strand, and only ATP6 and ATP8 overlap. The start codons were ATT (nine genes), ATG (three genes), and ATC (one gene), while all ended with a TAA stop codon.

The 16S rRNA and 12S rRNA were 1331 and 785 bp long with 83.6 and 82.7% AT content, respectively. The 22 tRNA genes ranged from 60 to 77 bp in size. TRNAscan-SE identified all tRNAs as folding into a typical cloverleaf secondary structure (Lowe and Eddy 1997). The CR was 570 bp long with a very high AT content (96.3%).

The phylogenetic position of *A. nuluensis* was determined using RAxML 8.2.0 (Stamatakis 2014) based on the concatenated nucleotide sequence of the 13 PCGs and two rRNAs genes. Support was assessed with 1000 bootstrap replicates. The tree topology showed that *A. nuluensis* clustered near *A. cerana* (Figure 1). Our findings are consistent with previously published molecular and morphological phylogeny data (Fuchs 1996). Furthermore, *A. nuluensis* clusters in a larger clade with *A. koschevnikovi* (also sympatric) and *A. nigrocincta* F. Smith (Figure 1), both Asian species of honey bees.

**Figure 1.**
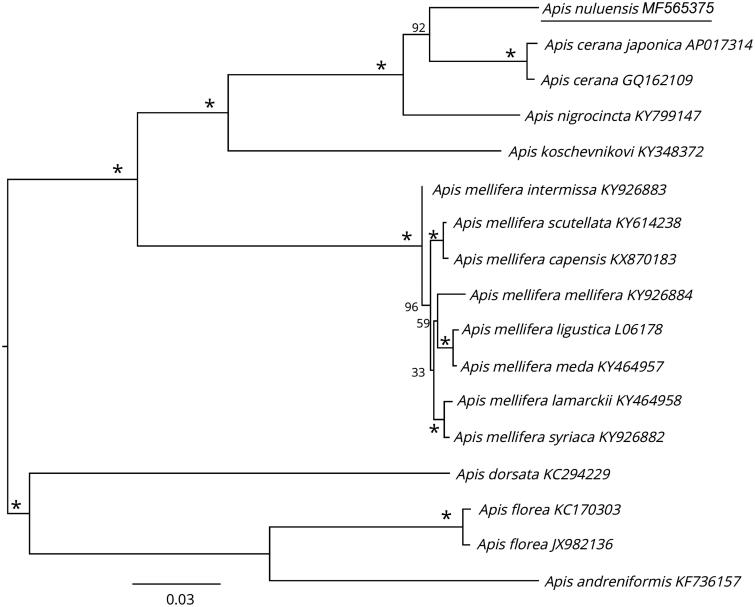
Phylogenetic relationship of *A. nuluensis* and other *Apis* species and subspecies based on concatenated dataset (13 PCGs + two rRNA genes) constructed by maximum likelihood approach. The GTR + G model was applied to each partition. Sixteen mitogenome sequences were obtained from GenBank and included in the tree. Numbers next to the nodes indicate bootstrap support. The GenBank accession numbers follow the scientific name. Star behind nodes indicate 100% bootstrap value.

The genetic divergences between *A. nuluensis* and *A. cerana*, *A. nuluensis* and *A. nigrocincta*, and *A. nuluensis* and *A. koschevnikovi* were 6.0%, 6.6%, and 12.0%, respectively. The genetic divergences between *A. nuluensis* and an allopatric subspecies of *A. mellifera* L. ranged from 12.9% (*A.m. intermissa* Buttel-Reepen) to 13.5% (*A.m. ligustica* Spinola). Comparisons using just *ND2* suggest a slightly greater genetic distance between the eight Asian *Apis* species and the one species occurring outside of Asia (18%; Arias and Sheppard 2005) than observed when using mitogenomic sequences. This *Apis* mitogenome possibly can be used to elucidate the evolutionary relationships among the *Apis* species and discover species-specific markers in *Apis*.
